# Longitudinal Cough Frequency Monitoring in Persistent Coughers: Daily Variability and Predictability

**DOI:** 10.1007/s00408-024-00734-x

**Published:** 2024-07-31

**Authors:** Kian Fan Chung, Carlos Chaccour, Lola Jover, Mindaugas Galvosas, Woo-jung Song, Matthew Rudd, Peter Small

**Affiliations:** 1 National Heart and Lung Institute, Imperial College London, Dovehouse St, London, SW3 6LY UK; 2 ISGlobal, Barcelona Institute for Global Health, Barcelona, Spain; 3 Clinica Universidad de Navarra, Pamplona, Spain; 4 Centro de Investigación Biomédica en Red de Enfermedades Infecciosas, Madrid, Spain; 5 Hyfe Inc., Wilmington, DE USA; 6 Department of Allergy and Clinical Immunology, Asan Medical Center, University of Ulsan College of Medicine, Seoul, South Korea; 7 University of the South, Sewanee, TN USA; 8 Department of Global Health, University of Washington, Seattle, WA USA

**Keywords:** Persistent cough, Chronic cough, Cough frequency, Artificial intelligence, Cough applications

## Abstract

**Purpose:**

We determined the cough counts and their variability in subjects with persistent cough for 30 days.

**Methods:**

The Hyfe cough tracker app uses the mobile phone microphone to monitor sounds and recognizes cough with artificial intelligence-enabled algorithms. We analyzed the daily cough counts including the daily predictability rates of 97 individuals who monitored their coughs over 30 days and had a daily cough rate of at least 5 coughs per hour.

**Results:**

The mean (median) daily cough rates varied from 6.5 to 182 (6.2 to 160) coughs per hour, with standard deviations (interquartile ranges) varying from 0.99 to 124 (1.30 to 207) coughs per hour among all subjects. There was a positive association between cough rate and variability, as subjects with higher mean cough rates (OLS) have larger standard deviations. The accuracy of any given day for predicting all 30 days is the One Day Predictability for that day, defined as the percentage of days when cough frequencies fall within that day’s 95% confidence interval. Overall Predictability was the mean of the 30-One Day Predictability percentages and ranged from 95% (best predictability) to 30% (least predictability).

**Conclusion:**

There is substantial within-day and day-to-day variability for each subject with persistent cough recorded over 30 days. If confirmed in future studies, the clinical significance and the impact on the use of cough counts as a primary end-point of cough interventions of this variability need to be assessed.

**Supplementary Information:**

The online version contains supplementary material available at 10.1007/s00408-024-00734-x.

## Introduction

Chronic cough affects up to 10% of the population worldwide [1, 2] and cough itself remains one of the most common complaints that drive patients to seek medical care [3]. Treatment targeted at putative causes does not often lead to its control [1]. The fundamental abnormality of refractory chronic cough is that of a chronic hypersensitivity state, secondary to neuroinflammation and possibly neural damage [4]. The impact of chronic cough has been assessed using patient-related outcomes such as Visual Analogue Scale (VAS) and Leicester Cough Questionnaires (LCQ) [5]. Although cough counting over a 24-h period has been available using semi-automated monitoring systems of the cough sound, it has not been used extensively in clinical practice [6]. Its greatest use has been in clinical trials of antitussive therapies, where one 24-h measurement is made before treatment and after treatment [7].

This restricted use of the 24-h cough monitoring has occurred because of the limitations of currently available systems using human annotation-based semi-automated cough counting systems [8]. The application of acoustic artificial intelligence (AI) and the ubiquity of smartphones have enabled the development of a system that continuously and unobtrusively monitor cough with preservation of privacy over periods of time longer than 1 day. Hyfe’s consumer cough tracking apps are smartphone wellness applications that detect coughs and allow users to record and review their own cough data [9, 10].

Chronic cough patients presenting in the cough clinic have highlighted the potential day-to-day or week-to-week variability in their cough patterns [8]. To understand this important and hitherto unexamined aspect of cough, we analyzed cough data collected from users of the Hyfe consumer app who monitored their cough over 30 days and measured the variability of cough frequencies, both within and between individuals.

## Methods

This is a retrospective observational study of data collected by Hyfe’s consumer cough tracking applications that are downloaded onto a user’s personal phone and, when active, run inconspicuously. They use the phone’s microphone to monitor sounds and recognize coughs with AI-enabled algorithms that run on the device. The precise times these coughs occur are uploaded via cellular network to an anonymized database maintained by Hyfe and the results are displayed on the phone as charts of coughs per hour. CoughTracker and CoughPro are consumer wellness apps available for download in the app stores (in the United States and outside the United States, respectively). They were freely available during the study period for both iOS and Android phones. Their availability was promoted through social media for use by individuals concerned about their cough. This study was conducted using data available to Hyfe with user consent under the user´s license agreement, which was digitally approved by each user, and was exempted from Institutional Review Board by the WCG ethics committee.

Between January 1 and August 4, 2023, 25,128 individuals downloaded and used Hyfe’s consumer apps at least once. The data obtained consisted of cough timestamps and app usage times but do not include identifying, demographic or clinical information about the users. To study individuals with persistent cough, we filtered this dataset to identify those users who (a) monitored their cough for 20 h or longer on at least 30 different (but not necessarily consecutive) days and (b) had at least 5 coughs per hour on average per day; this threshold mitigates the possible contamination of cough frequency estimates by false positives.

### Analysis of Cough Frequency and Predictability

Hourly cough counts were tabulated for each subject over the 30 days: for each day, the number of coughs was divided by the total monitoring time to calculate the day's cough frequency. We used a resampling analysis of the 20 to 24-h cough counts from each day to calculate a 95% confidence interval estimate of cough frequency. This yields cough counts and confidence intervals for every user for each of the 30 days.

The accuracy of any given day for predicting all 30 days is the *One Day Predictability* for that day, calculated as the percentage of days where cough frequencies fell within that day’s 95% confidence interval. Each user’s *Overall Predictability* was then defined as the mean of the 30-One Day Predictability percentages. This summarizes the ability of one day of cough data to predict the daily cough frequency on other days. A person with a stable, predictable cough pattern and high coverage percentages do not change much from day-to-day, while a person with an unstable cough pattern will have low coverage percentages that varied more from 1 day to the next.

## Results

The eligible pool of subjects consisted of 25,128 individuals who downloaded and used Hyfe’s consumer apps at least once between 1st January and 4th August 2023 (Supplementary Figure S1). 213 users monitored for at least 20 h per day on at least 30 days, and of these subjects, 97 users had cough frequencies of at least 5 coughs per hour.

### Daily Cough Variability Within and Between Subjects

**Fig. 1:**
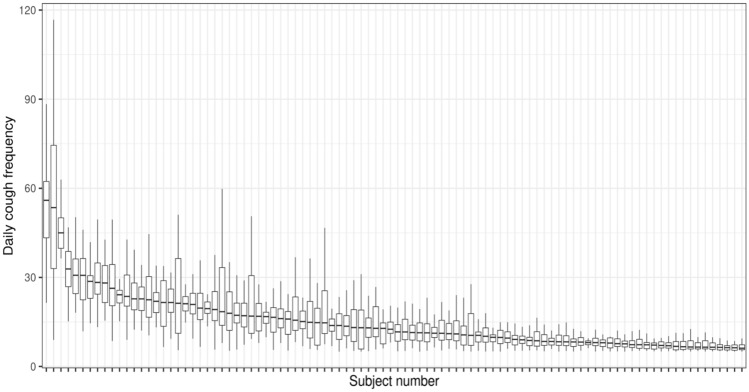
Boxplots of the daily cough frequencies over the period of observation of 97 individuals who monitored for at least 30 days and had frequencies of over 5 coughs per hour. Subject 80, an outlier with a median frequency of 160.08 coughs per hour, is not shown. For each subject, the frequency quartiles are shown by the bottom edge (first quartile), midline (median), and top edge (third quartile) of the box; the range is depicted by the vertical lines. Cough rates vary considerably among this cohort, both within and between subjects

**Fig. 2:**
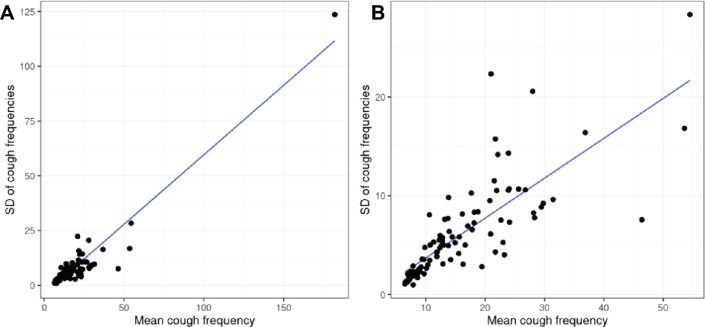
Standard deviations (SD) and means of daily cough frequencies of 97 individuals who monitored for at least 30 days and had frequencies of over 5 coughs per hour. Each point represents one subject; the x-coordinate is the average of that subject’s daily cough frequencies, and the y-coordinate is the SD of that subject’s daily cough frequencies. Panel **A** shows all 97 individuals (correlation = 0.95; Optimized Least Square (OLS) slope = 0.64), while Panel **B** shows 96 subjects with the exclusion of Subject 80 (correlation = 0.79; OLS slope = 0.40). Lines of best fit are shown

The variability in cough within and between subjects can be seen in Figs. 1 and 2, with the median cough frequencies varying from 6.17 to 55.96 coughs per hour. The individual mean cough rates exhibit similar variability, ranging from 6.48 to 54.51 coughs per hour. Subject 80, not shown in Fig. 1, had a median (mean) cough frequency of 160.08 (182.04) coughs per hour.

Figure 1 demonstrates the variability of cough frequencies within individuals, since the vertical span of each boxplot provides the range of cough frequencies for that one subject. Quantitatively, the IQRs for these subjects’ cough frequencies vary from 1.30 to 41.48 coughs per hour, with SDs (shown in Fig. 2) ranging from 0.99 to 28.35 coughs per hour. (Subject 80’s IQR and SD were 206.95 and 123.62, respectively.)

### Daily Cough Frequency Predictability

**Fig. 3:**
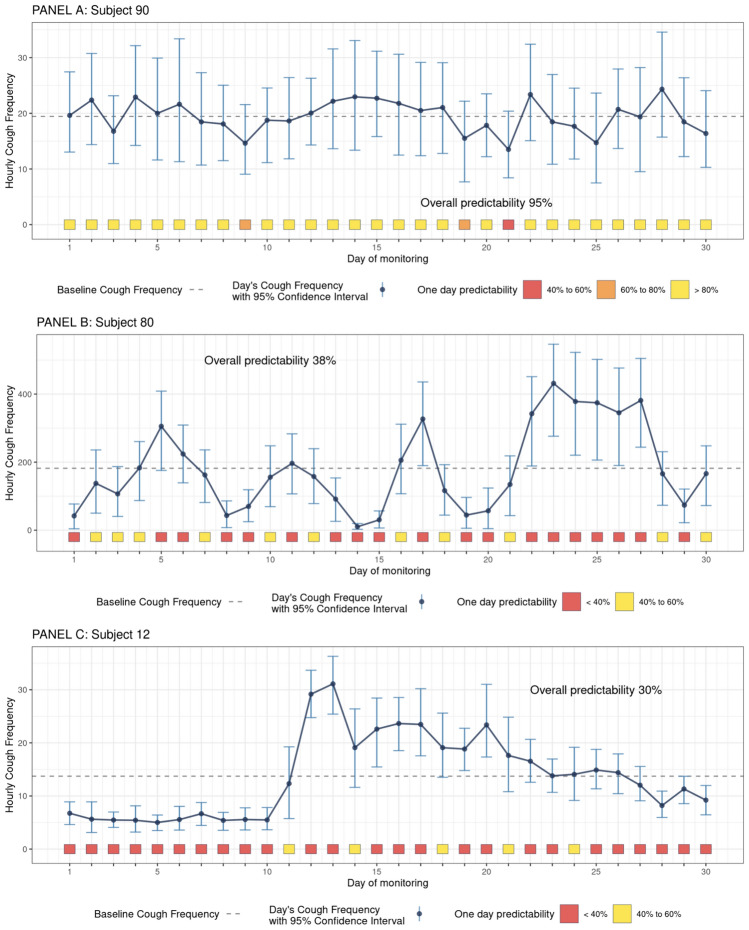
Daily cough frequencies data are shown as dots with the 30 day average depicted by the dashed line. The 95% confidence interval of cough frequency for each day is represented by the vertical bars. The coverage percentage for each day is shown at the bottom (color coded as yellow for > 80%, darker for between 60 and 80%, darker for between 40 and 60% and red for < 40%). In Panel **A**, the user’s cough frequency predictability is 95%, in Panel **B** subject this is 38% and in Panel **C**, for this subject 30%

The large intra-subject variability has implications for how well any day’s results predict the entire time-period. We detail the calculation for two extreme examples of the 1 day and overall predictability and one subject with a deterioration in coughing (Fig. 3). The cough data from the subject with the most consistent daily cough rates (Number 90) is shown in the top panel of Fig. 3. On the first day of monitoring, the user had a cough frequency of 19.6 coughs per hour and a 95% confidence interval of 13.0 to 27.4, just slightly above their 30-day daily cough frequency average of 19.4 coughs per hour. Based on this first day of data, we would predict this user’s other daily cough frequencies to fall between 13.0 and 27.4 coughs per hour, which was accurate for all 30 days of this user’s observed cough frequencies. Thus, the One Day Predictability of the first day was 100%. However, on day 21, this user had a cough frequency of 13.5 coughs per hour and a 95% confidence interval of 8.4 to 20.4 coughs per hour, an interval that only captured the daily cough frequencies of 18 of the 30 days yielding a one day predictability of 60%. Averaging the 30-one day predictabilities over all 30 days yields an overall predictability of 95%. This subject demonstrates that 24-h cough counts can be predictive when cough rates are stable.

In contrast, the middle panel of Fig. 3 shows the cough data for a user with a more variable cough rate (Subject 80). On the first day of monitoring, the user had a cough frequency of 42.0 coughs per hour and a 95% confidence interval of 4.3 to 77.1. Based on this first day of data, we would predict this user’s other daily cough rates to range between 4.3 and 77.1, which was accurate on only 8 of this user’s other daily cough rates, yielding a One Day Predictability of 26.7% percent. Averaging the 30-one day predictabilities for this subject yields an overall cough predictability of only 38%. This case exemplifies the fundamental problem with limited cough monitoring in individuals with volatile cough rates.

Subject number 12 has a cough pattern that suggests a period of stability followed by a deterioration in cough that gradually resolves (bottom panel of Fig. 3). During the first 10 days of monitoring, this user’s daily cough frequency was about 5 coughs per hour, then increased to 31 coughs before gradually returning towards baseline over the subsequent days. On the first day of monitoring, the cough rate was 6.75 coughs per hour. Despite accurately predicting the stable period of the first 10 days, given its poor performance for the remaining 20 days, the One Day Predictability for this day is only 30%. The One Day Predictability on day 13, when an increase in cough frequency occurred, is an abysmal 6.7%, after which the One Day Predictability varies from 13.3% (day 28) to 53.3% (day 11). The overall predictability for this subject is 30.4%. The average 30-day cough frequency of 13.7 coughs per hour is a meaningless summary, as they experienced two very different cough patterns demarcated by day 12. This demonstrates the value of continual cough frequency monitoring in identifying individuals with increases in coughing.

### Variability of Daily Cough Predictability

**Fig. 4:**
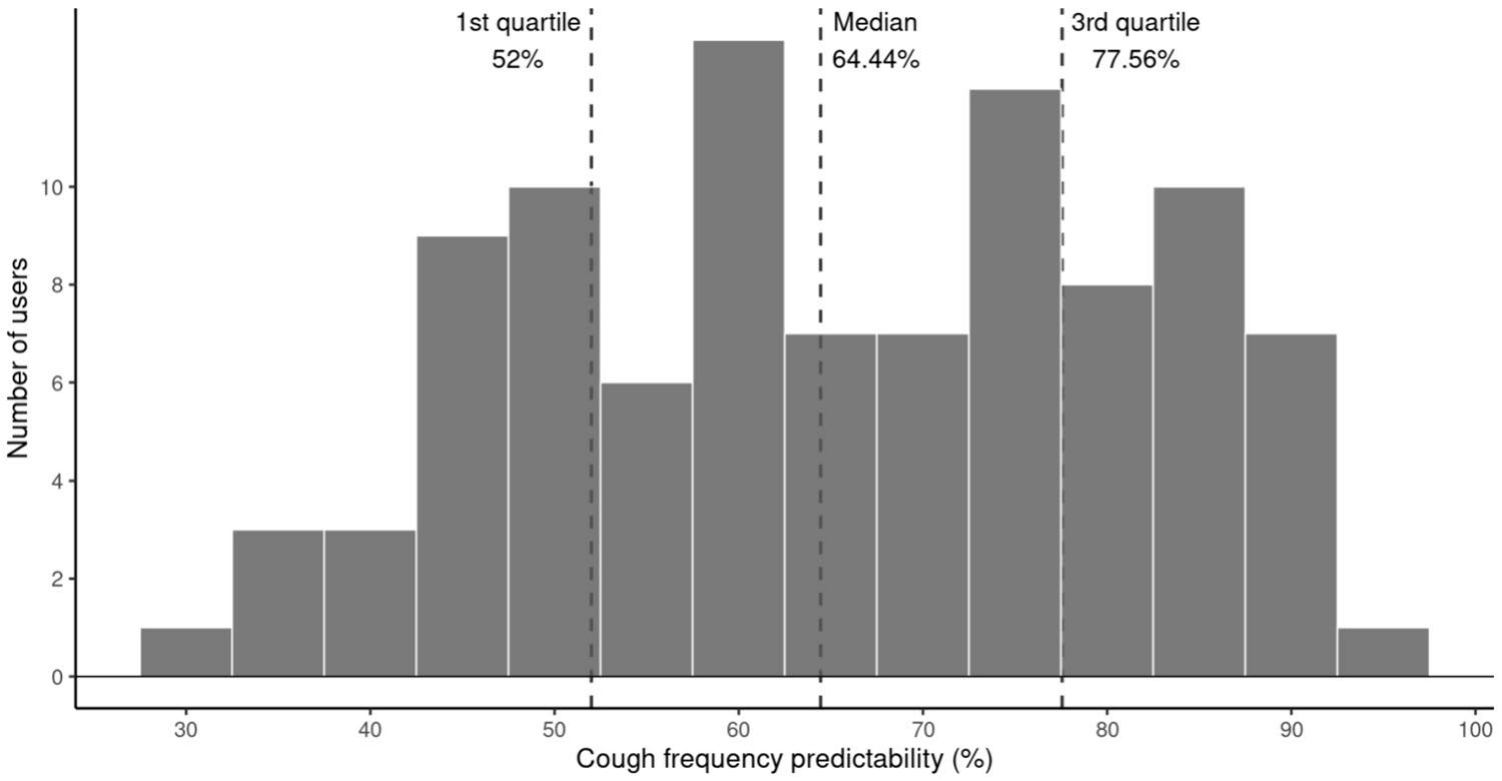
Distribution of overall cough frequency predictabilities of the 97 subjects

**Fig. 5:**
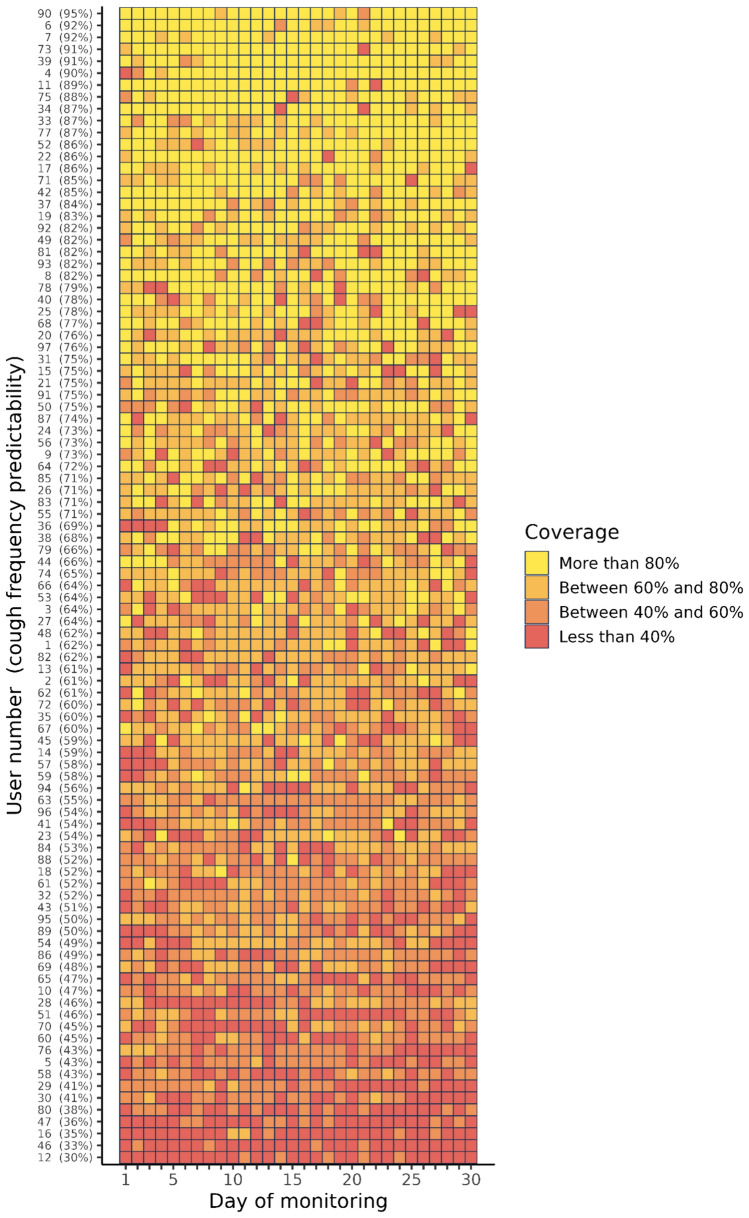
Daily coverage of daily cough frequency predictability results for all users. Each row represents one user and each cell is one day. The color of each cell corresponds to the coverage percentage for that day (yellow for > 80%, slightly darker for between 60 and 80%, darker for between 40 and 60%, and red for < 40%)

Overall cough frequency predictability varied markedly among the 97 users (Fig. 4). Figure 5 summarizes the daily coverage results for all users: each row is one individual, each cell is 1 day, and the color of the cell indicates the coverage for that day. The rows are ordered by cough frequency predictability, with user 90 at the top, as the most stable and predictable cougher, and user 12 at the bottom, as the least stable and predictable cough subject. For users at the top of this plot, one day of cough data yields an accurate prediction of the daily cough frequency for the entire period. By contrast, the bottom of this plot demonstrates that one day of cough data does not accurately predict daily cough frequency on days other than the one being measured.

The cough frequency time series for all 97 users are provided in Supplementary Figure S2.

## Discussion

Out of 25,128 individuals who downloaded and used Hyfe’s consumer app at least once during a 7-month period, 97 users monitored for at least 20 h per day on 30 or more days and had cough frequencies of at least 5 coughs per hour. Because we have developed a system by which cough could be identified instantaneously and by which cough can be recorded continuously over days, we can be confident that the 97 users had a persistent cough, i.e., a cough that occurred on a daily basis. Chronic cough is defined on the basis of longevity usually of 8 weeks [11] or 12 weeks [12] for adults, and of 4 weeks in children [13], and thus we were unable to confirm in these 97 adults whether they had chronic cough by definition. However, if the recordings had continued for 8 or 12 weeks, we would be confident that these subjects would indeed be subjects with a chronic cough. Hence, this app could be used to confirm a diagnosis of chronic cough in those presenting in a chronic cough clinic. However, as all of the data collected was completely anonymized, we can only deduce that these subjects have a persistent problematic cough without any indication of the cause of their cough or knowledge of how representative they are of chronic coughers.

A wide variation of daily cough counts can exist between these subjects, and also on the different days within the same subject. Within this group, the mean daily cough rate was positively associated with the standard deviation indicating that the higher the mean cough rate, the higher the variability of cough rates over the 30 days of measurement. Furthermore, for the analysis of the day-to-day variability, we calculated the Overall Predictability for each subject by determining the ability of each day’s counts to predict longer term counts, which also varied between different subjects from 30 to 95%. Someone with a stable cough pattern will exhibit predictable, random fluctuations in cough frequency, but someone with a volatile cough pattern will have fluctuations around an average that is itself changing and unpredictable.

In the 97 subjects, only one third of subjects had an Overall Predictability of 75% or higher, reflecting a stable daily cough pattern such that 1 day of monitoring would be sufficient to describe a subject’s cough. The bottom third of subjects had an Overall Predictability of 60% or lower; for them, the variability in their cough is unpredictable and longer periods of monitoring would be needed to have a more correct description of their daily cough rates. If confirmed in future studies, this comes into serious consideration in clinical trials of novel antitussives as to the selection of patients with chronic cough for these trials. These trials would need to use more prolonged cough recordings than just a 24-h recording at the start and another 24-h recording at the end of the trial. The use of longer cough recording periods would in itself need new ways of analyzing such data as just one day’s average cough count is not enough to characterize a chronic cougher’s cough pattern. Thus, our data and that of the data we have collected in patients with chronic cough attending a cough clinic [14] would firmly support that such longitudinal cough monitoring should be used in both clinical care and cough science research. Such work will provide fundamental insights into the statistics of cough and strengthen the design and implementation of clinical trials of novel antitussives.

This large variability in cough count between subjects with chronic cough and also on different days within subject deserves some explanation and investigations. We hypothesize that it is unlikely that this is due to an inherent fault in the system for detecting cough sound given the robustness of the Hyfe algorithms. The variability brings up the possibility that the subject with chronic cough may be exposed variably to different amounts of triggers during his/her daily activities; alternatively, it is possible that there is a diurnal and day-to-day variability of the underlying cough hypersensitivity that underlies chronic cough [4]. Recording the cough events in association with the subjects’ activity and position may help in determining the potential triggering factors in these individuals with a cough hypersensitivity.

The limitations of this study stem from the subjects being an anonymized convenience sample obtained from subjects on whom we do not have clinical information and the use of a largely unvalidated app run on the users mobile phones for monitoring. Nevertheless, given their voluntary and persistent cough monitoring for weeks which has been confirmed by the AI-instructed app, we can conclude that these users represent a subpopulation of people with problematic persistent cough, the population from which cough study and antitussive trial subjects are recruited. We have analyzed data collected solely from Hyfe’s consumer cough tracking apps. As such, a user can be far from their phone while running the app, thereby missing coughs that would otherwise be detected. It is also possible that the cough tracking app might record ambient coughs from others in the environment as that of the subject. This risk is mitigated because the Hyfe app is optimized to detect coughs within a one meter radius. Even if a few coughs from nearby individuals are detected, given the high cough rates, they are not likely to significantly impact the accurate measurement of the primary user's cough rate.

This study provides important guidance regarding subject selection, monitoring technology and study design for future longitudinal clinical studies of cough. Specifically, there would be value in studies of well defined cohorts for a variety of conditions. It would be important in future studies to use cough monitors that have been validated to passively and continually monitor coughs and that can detect when monitoring has been interrupted. Finally, studies should note the times of cough in he local time zone and also record key aspects of the users behavior, such as bedtime or consequences of coughing such as urinary incontinence.

In conclusion, the cough frequency variability with good to poor levels of predictability demonstrated in people with chronic cough supports the necessity for longitudinal cough monitoring over many days. If this is confirmed in future studies, it demonstrates that 1 day of monitoring is often insufficient to describe a person’s cough accurately; even when 1 day of data is enough, that fact can only be verified by monitoring for many days. This conclusion is in support of the recent guidelines on chronic cough that advocates the need for a fully automated cough recording technology that continuously monitors patients in real-time [15].

## Supplementary Information

Below is the link to the electronic supplementary material.

Supplementary file1 (DOCX 5203 KB)

## Data Availability

No datasets were generated or analyzed during the current study.
