# Supplementary material for: Longitudinal Cough Frequency Monitoring in Persistent Coughers: Daily Variability and Predictability
Source: Lung. 2024 Jul 31;202(5):561–8. doi: 10.1007/s00408-024-00734-x (PMC11427503; doi:10.1007/s00408-024-00734-x)

**Longitudinal cough frequency monitoring in persistent coughers:**

**Daily Variability and Predictability**

Kian Fan Chung^1^*, Carlos Chaccour^2, 3, 4^ , Lola Jover^5^ , Mindaugas Galvosas^5^,

Woo-jung Song^6^, Matthew Rudd^5, 7^, Peter Small^5, 8^

^1^National Heart and Lung Institute, Imperial College London, London, UK.

^2^ ISGlobal, Barcelona Institute for Global Health, Barcelona, Spain;

^3^ Clinica Universidad de Navarra, Pamplona, Spain

^4^ Centro de Investigación Biomédica en Red de Enfermedades Infecciosas, Madrid, Spain

^5^ Hyfe, Wilmington, DE, USA;

^6^ Department of Allergy and Clinical Immunology, Asan Medical Center, University of Ulsan College of Medicine, Seoul, South Korea

^7^ University of the South, Sewanee, TN, USA

^8^ University of Washington, Department of Global Health, Seattle, WA, USA and Hyfe

**Supplementary data**

Supplementary Figure S1

Supplementary Figure S2

**Supplementary figure S1:**

Flow diagram for inclusion of study subjects from initial pool of users

**
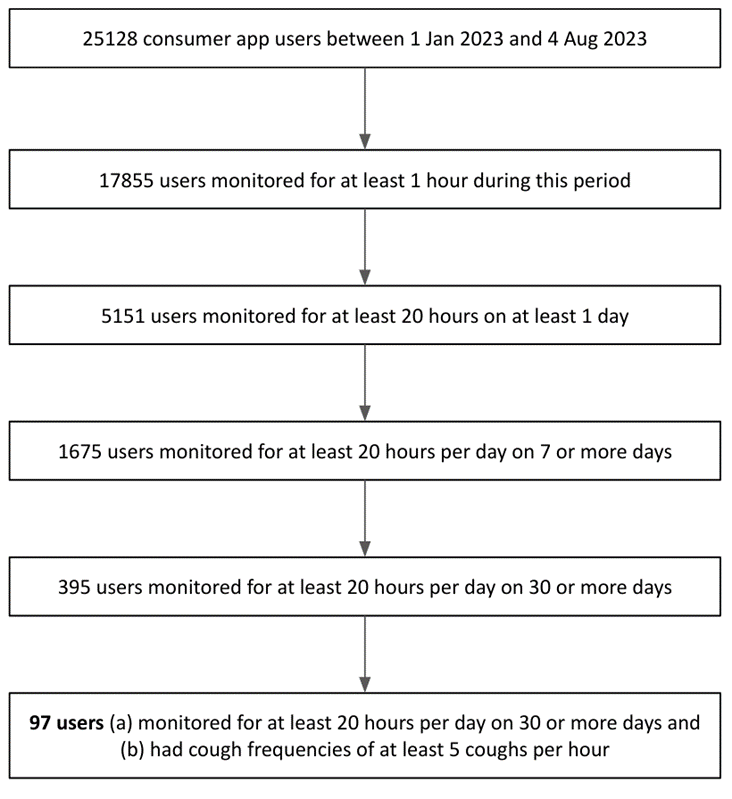
**

**Supplementary Figure S2**:

Daily cough frequency time series for all 97 subjects ordered by cough frequency predictability as documented in Figures 1, 2 and 3, from the more predictable to the less predictable. The y axis is coughs per hour and the x-axis represents 30 successive days.


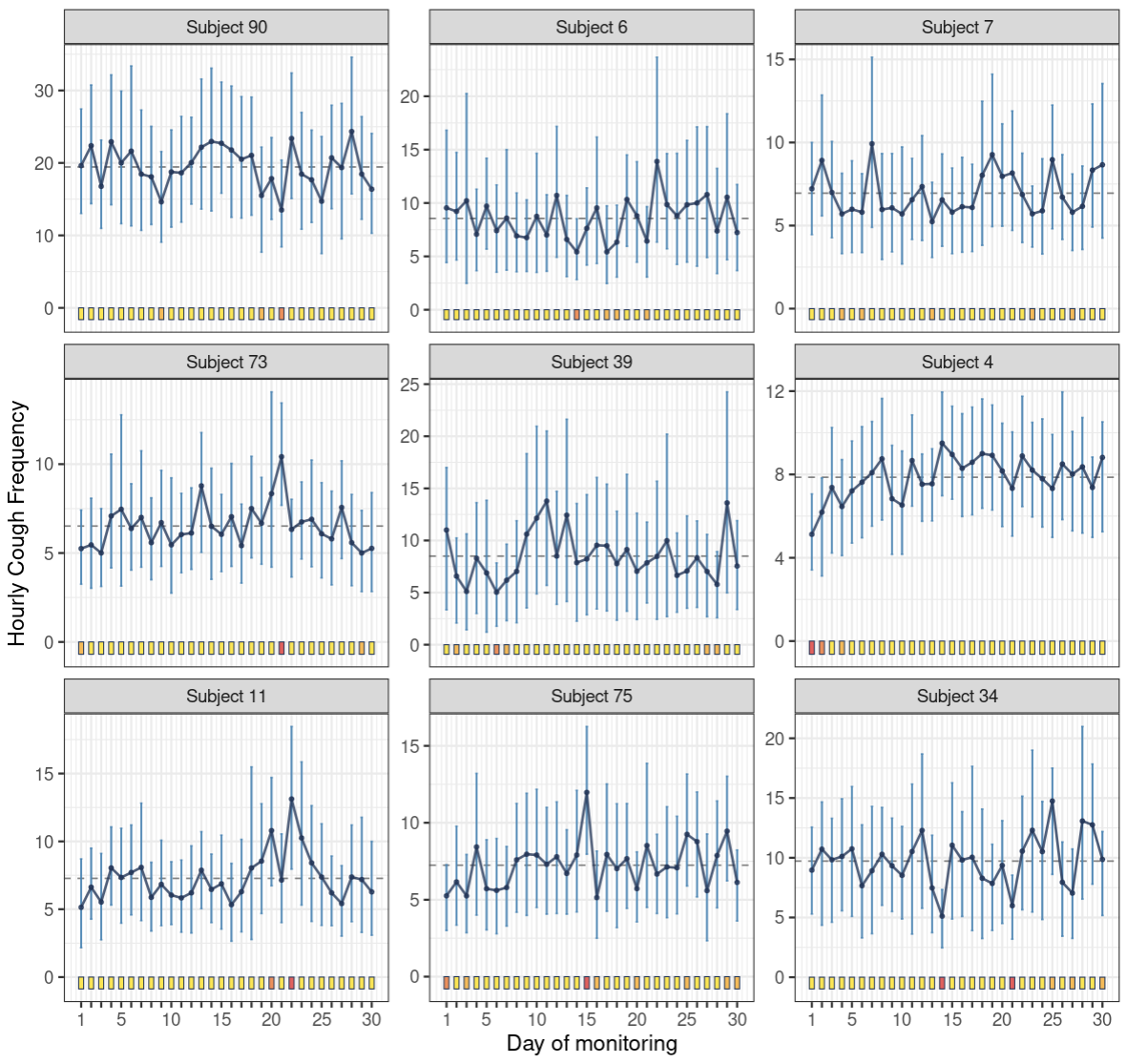


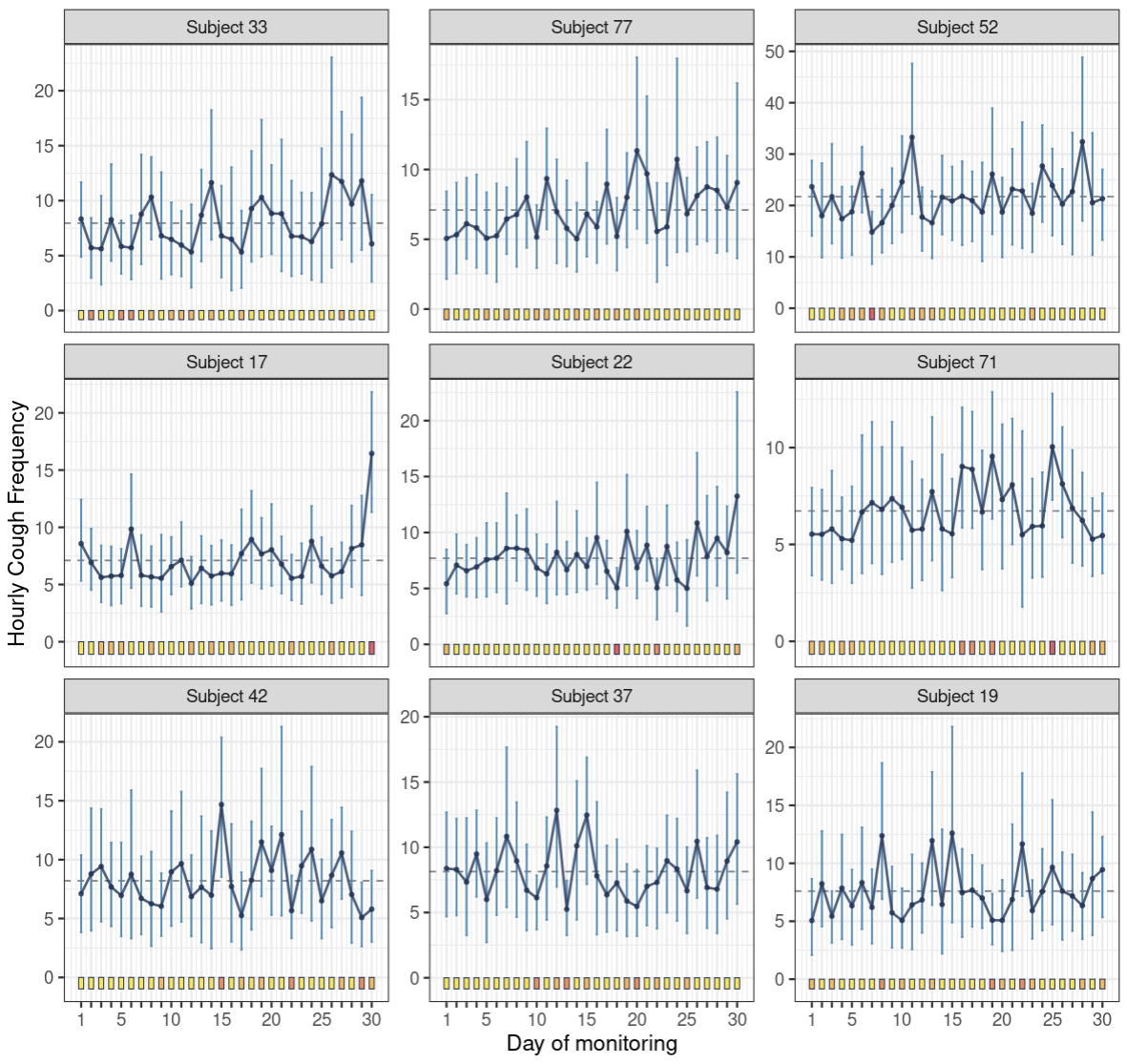


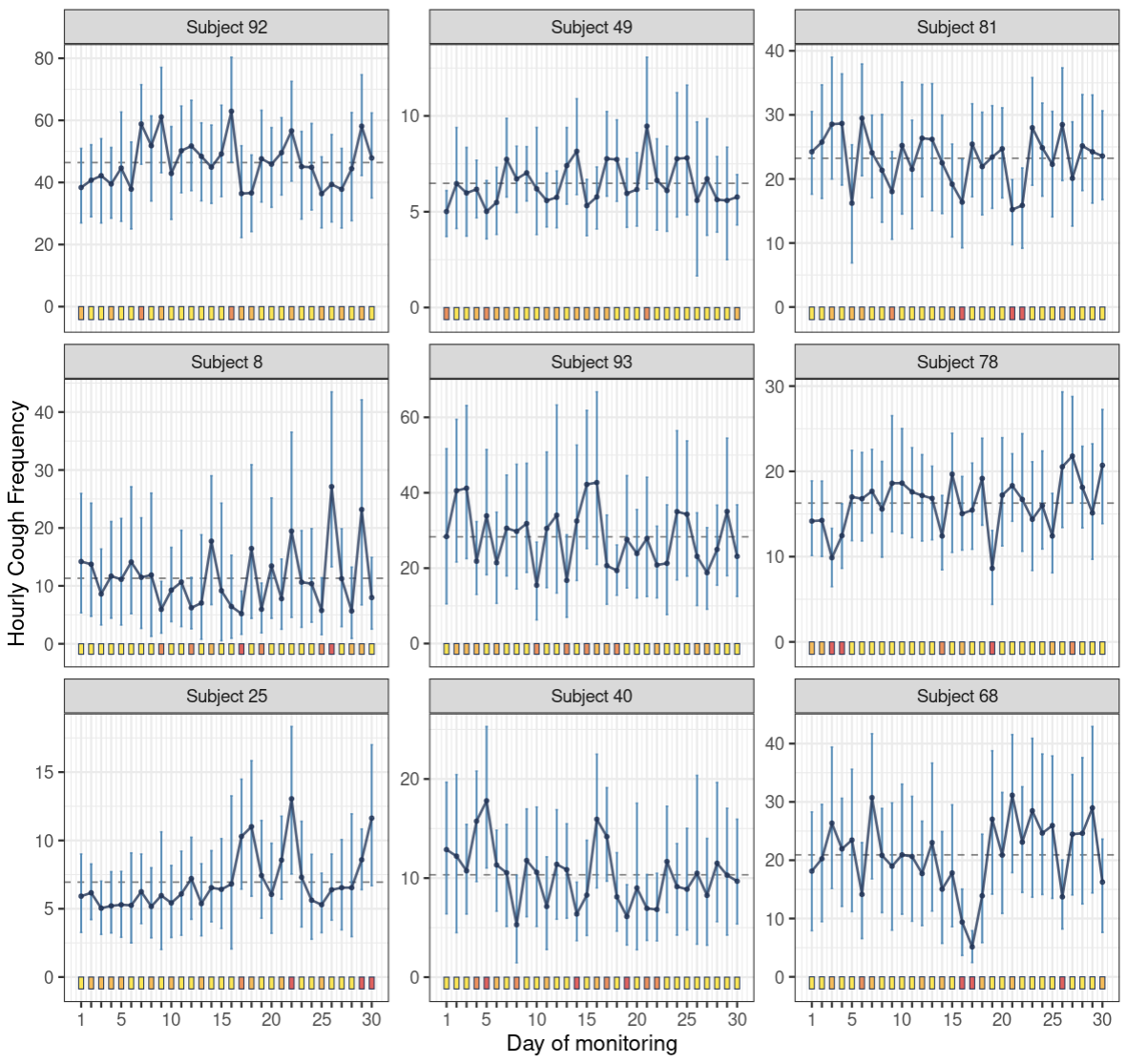


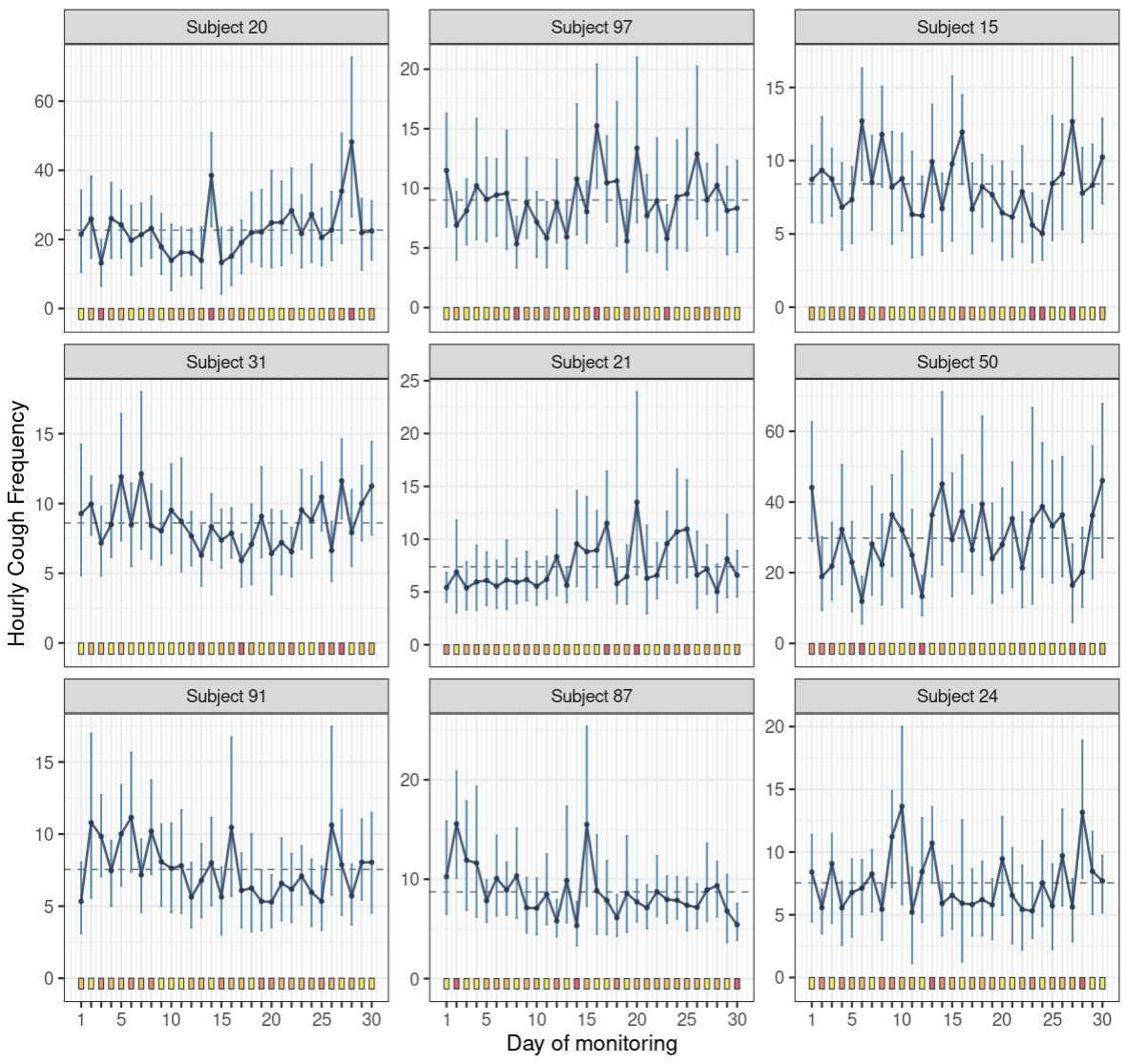


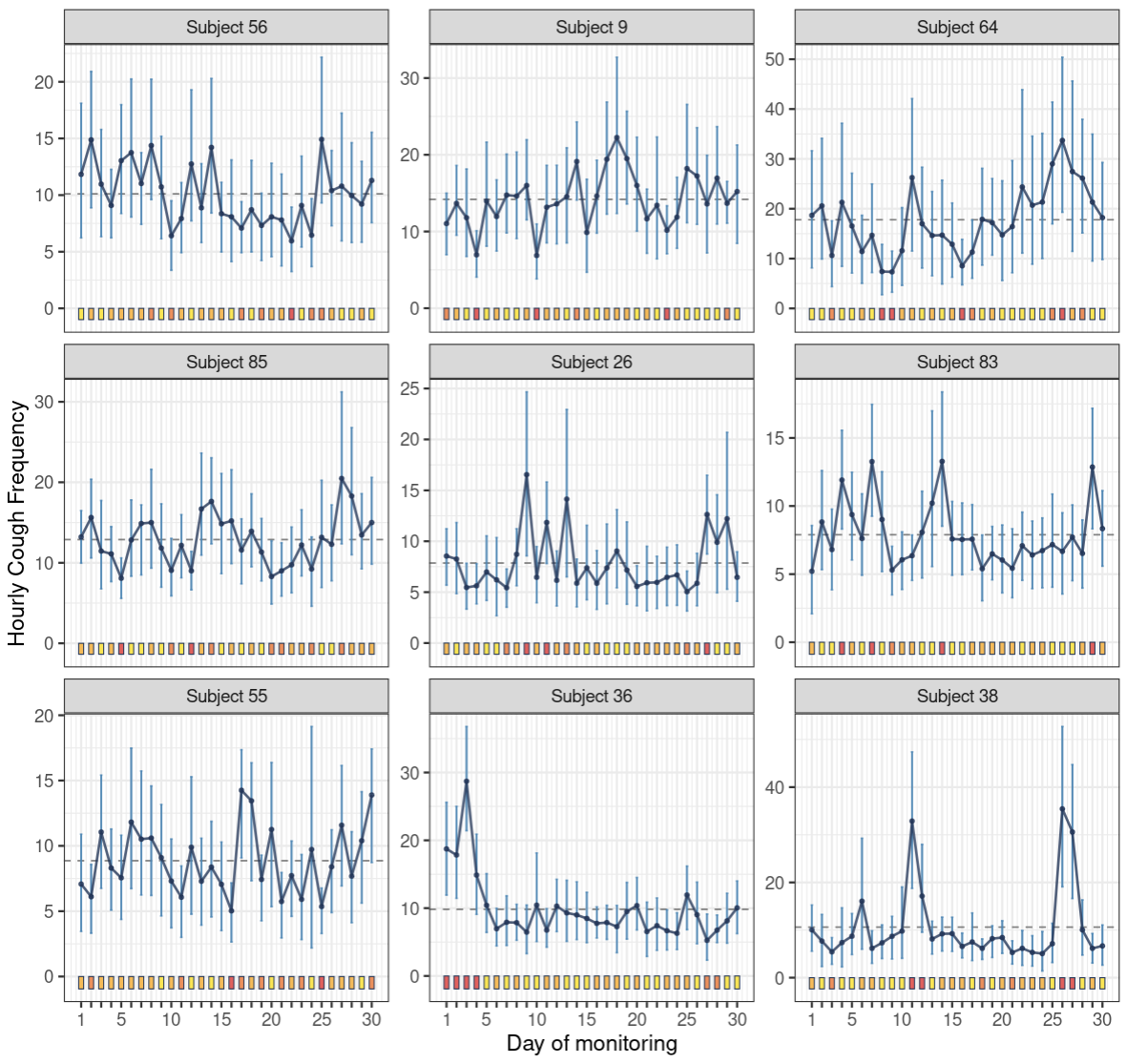


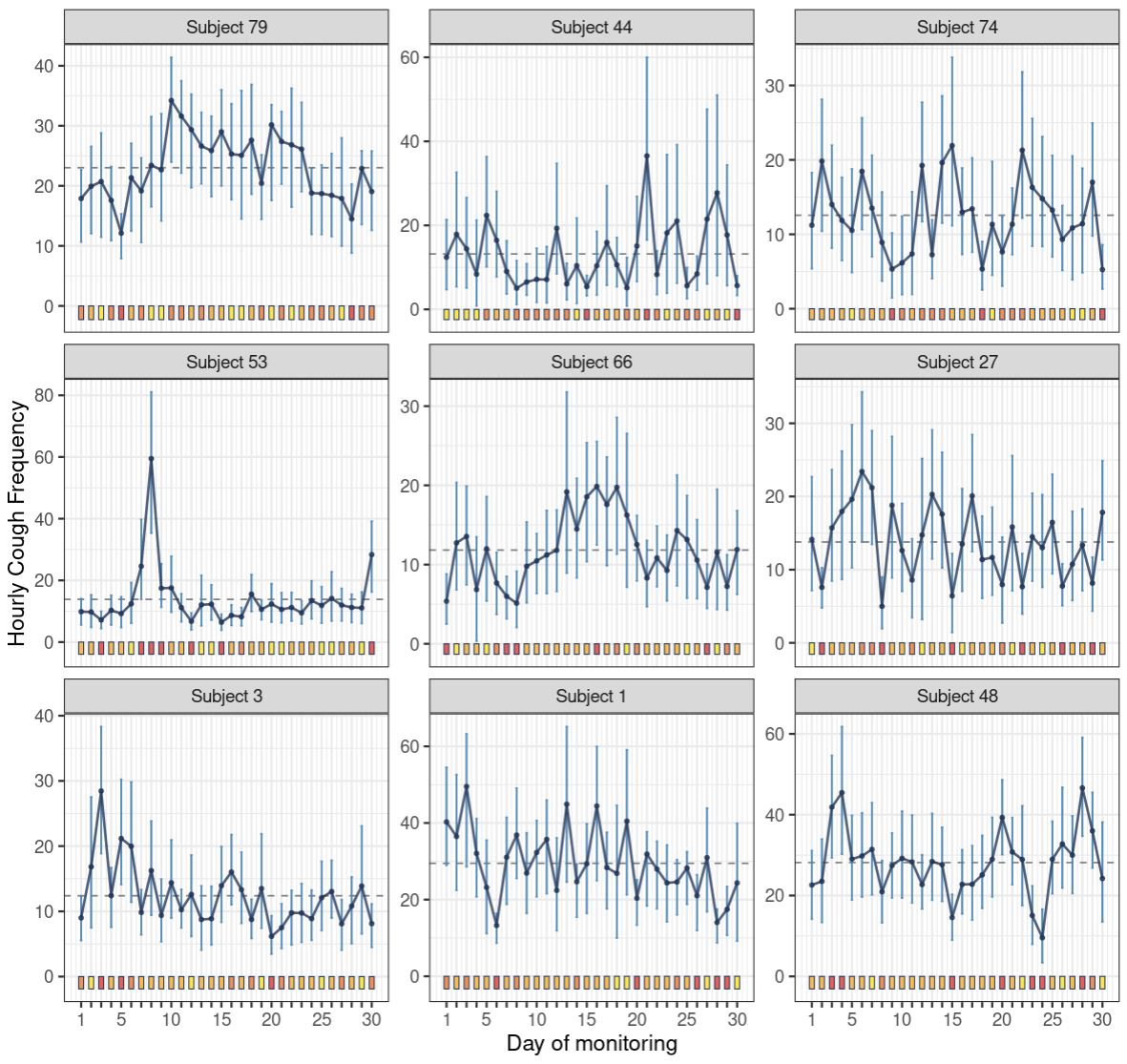


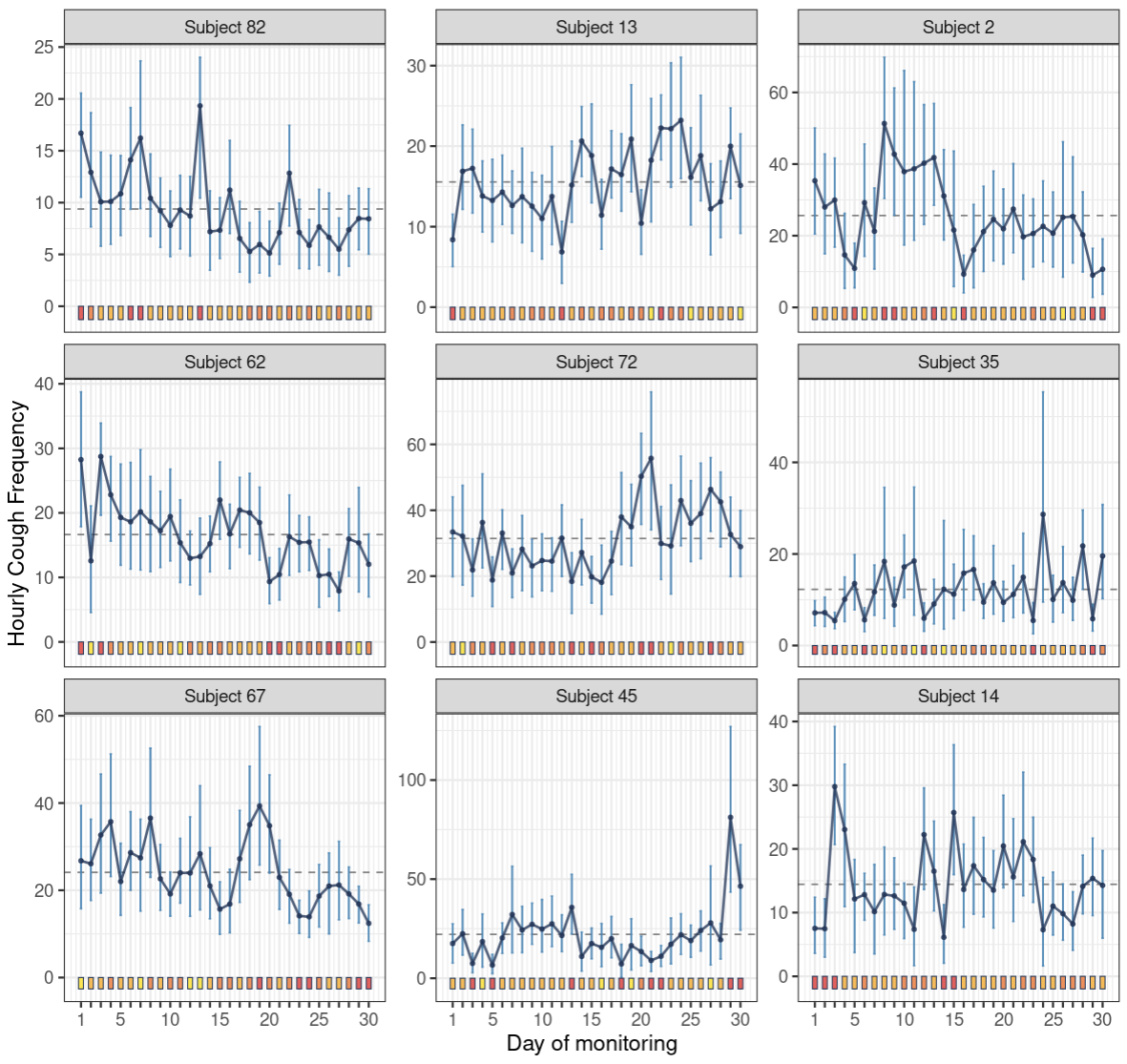


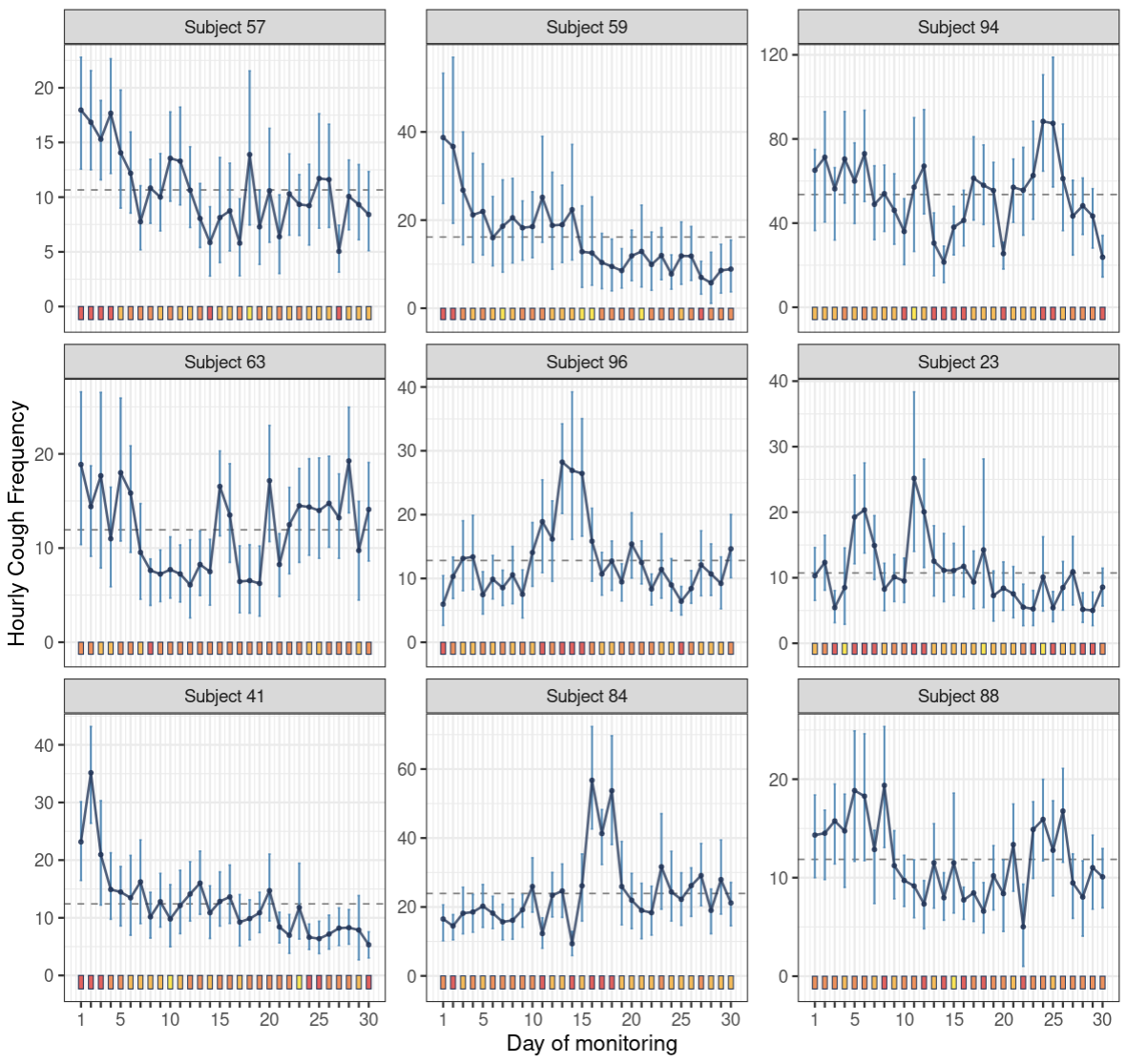


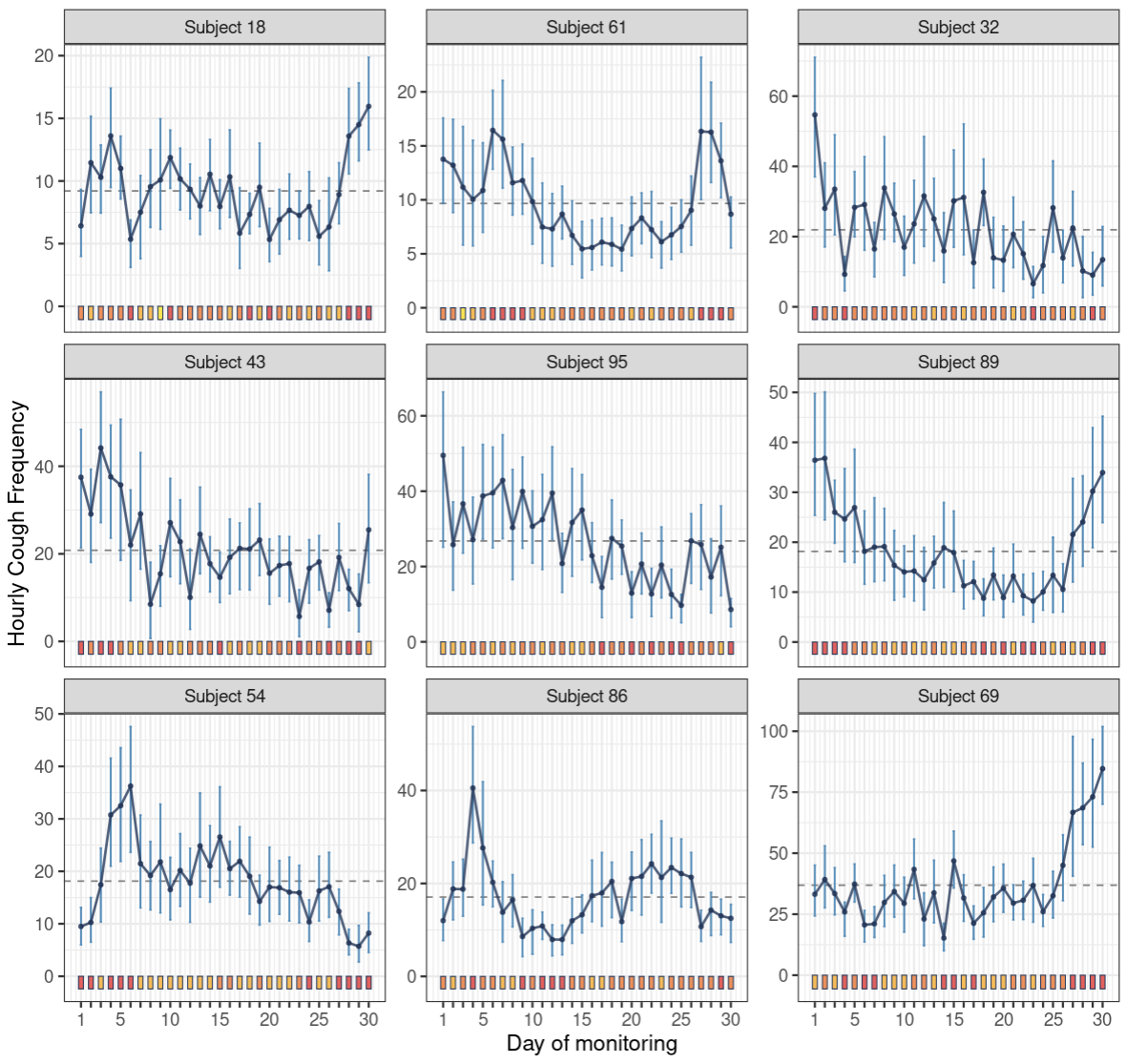


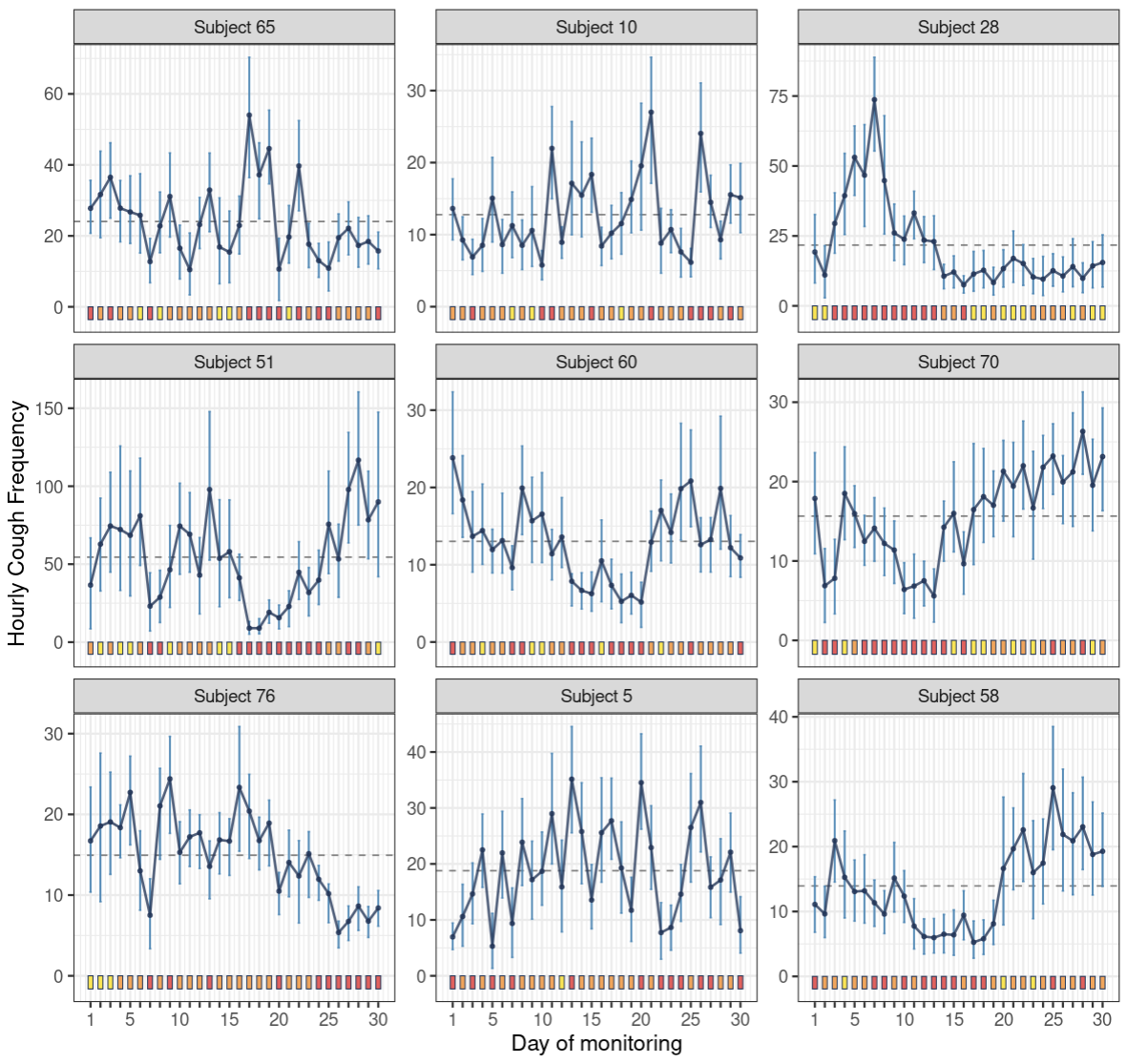


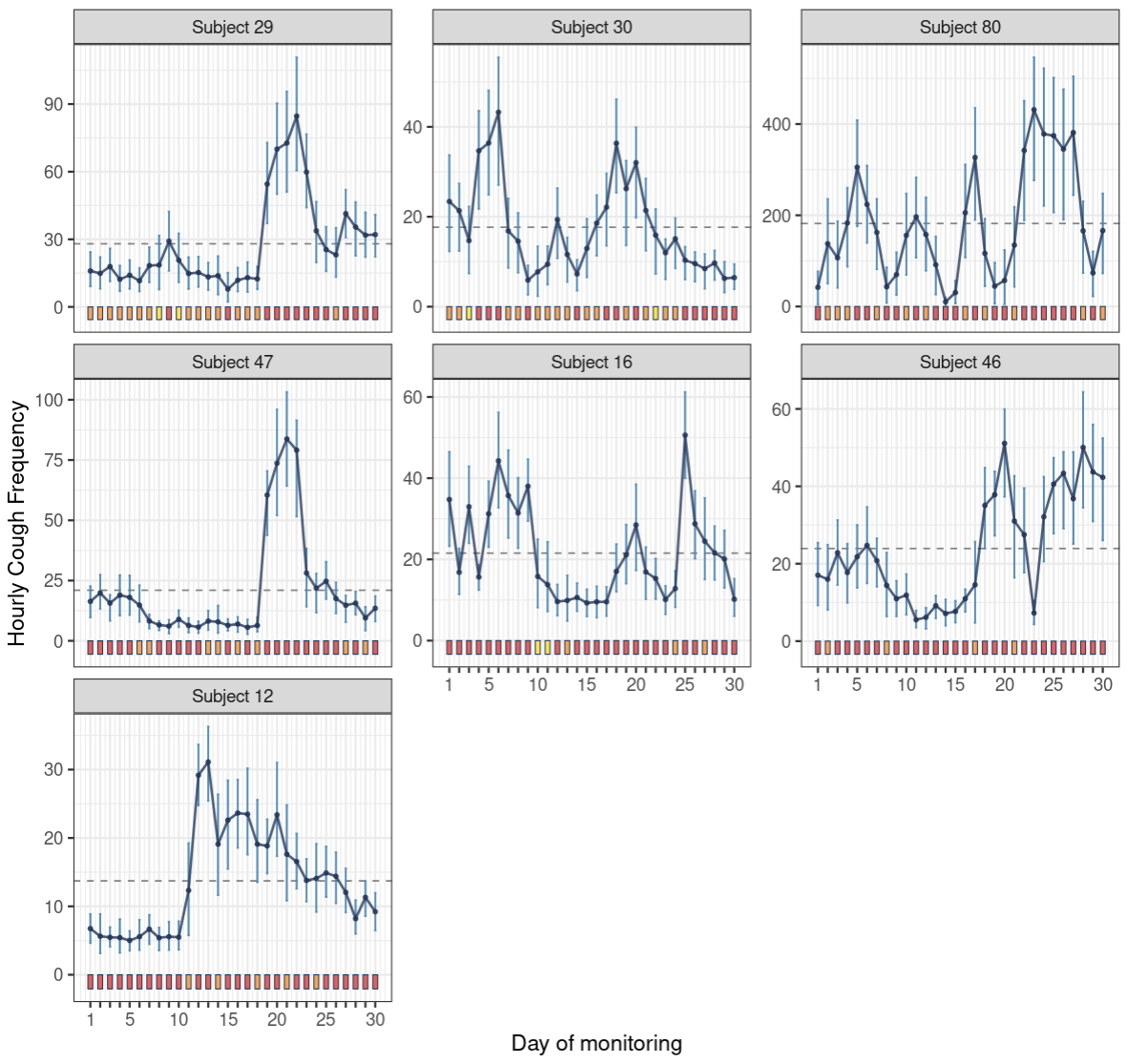

Supplement: Supplementary file 1 — Supplementary file1 (DOCX 5203 KB) [file 408_2024_734_MOESM1_ESM.docx]
